# Biomarkers in Cerebrospinal Fluid for the Diagnosis and Monitoring of Gliomas

**DOI:** 10.3390/biom14070801

**Published:** 2024-07-05

**Authors:** Dimosthenis Papadimitrakis, Miltiadis Perdikakis, Antonios N. Gargalionis, Athanasios G. Papavassiliou

**Affiliations:** 1 Department of Biological Chemistry, Medical School, National and Kapodistrian University of Athens, 11527 Athens, Greece; dipapadimitrakis@gmail.com (D.P.); miltiper@gmail.com (M.P.); 2 Laboratory of Clinical Biochemistry, Medical School, ‘Attikon’ University General Hospital, National and Kapodistrian University of Athens, 12462 Athens, Greece

**Keywords:** biomarker, cerebrospinal fluid, ctDNA, extracellular vesicle, glioma, miRNA

## Abstract

Gliomas are the most common type of malignant brain tumor and are characterized by a plethora of heterogeneous molecular alterations. Current treatments require the emergence of reliable biomarkers that will aid personalized treatment decisions and increase life expectancy. Glioma tissues are not as easily accessible as other solid tumors; therefore, detecting prominent biomarkers in biological fluids is necessary. Cerebrospinal fluid (CSF) circulates adjacent to the cerebral parenchyma and holds promise for discovering useful prognostic, diagnostic, and predictive biomarkers. In this review, we summarize extensive research regarding the role of circulating DNA, tumor cells, proteins, microRNAs, metabolites, and extracellular vesicles as potential CSF biomarkers for glioma diagnosis, prognosis, and monitoring. Future studies should address discrepancies and issues of specificity regarding CSF biomarkers, as well as the validation of candidate biomarkers.

## 1. Introduction

Gliomas are the most common malignancy in the central nervous system (CNS). They originate from glial cells that support neurons and include ependymomas, astrocytomas, oligodendrogliomas, and glioblastomas. They are distinct but challenging entities in the field of CNS neoplasms. As imaging techniques cannot distinguish different neoplastic entities, glioma diagnosis usually requires a biopsy of the tumor tissue after surgical removal. Therefore, less invasive methods are needed for the diagnosis of gliomas. Liquid biopsy, the sampling and analysis of body fluids, may be a valuable and reliable tool for detecting and monitoring these tumors. Methods used for the detection of tumor biomarkers in liquid tissue include enzyme-linked immunosorbent assay (ELISA), polymerase chain reaction (PCR), and next-generation sequencing (NGS). For CNS malignancies, cerebrospinal fluid (CSF) has emerged as an optimal candidate and contains significantly greater levels of CNS tumor biomarkers than any other body fluid [1,2,3].

The present article provides a comprehensive overview of the characteristics, classification, and molecular underpinnings of gliomas, setting the stage for a detailed exploration of CSF biomarkers, including circulating tumor DNA (ctDNA), microRNAs (miRNAs), circulating tumor cells (CTCs), exosomes, and proteins. Understanding these molecular signatures in CSF holds significant promise for the noninvasive detection and monitoring of gliomas, potentially influencing treatment decisions and improving patient outcomes.

## 2. Types of Gliomas and Molecular Pathogenesis

### 2.1. Glioblastoma

Glioblastomas (GBMs) represent the most common type of malignant brain tumor. They are classified as grade 4 gliomas by the World Health Organization (WHO) and have a median survival of only 15 months following therapeutic approaches (surgery and chemoradiation therapy). The 5-year survival of patients suffering from GBM is less than 5%, indicating a poor prognosis. GBM can affect people of all ages (median age is usually cited to be in the 60-year range), but it is most common between ages 75–84 [1]. The only environmental factor successfully associated with the development of GBM is ionizing radiation, especially after years of radiotherapy for different tumors [2]. GBM cells are derived from adult neural stem cells, progenitor cells (NSPCs), and glioma-initiating cells. Tumors are mostly in the frontal, temporal, and parietal lobes [3,4]. Metastasis is rare. The clinical presentation of GBM is atypical and depends on the area of the brain affected and the size and stage of the tumor. During the clinical course of the disease, symptoms such as headache, numbness, loss of vision, mood, memory disorders, nausea, vomiting, fatigue, sensorimotor deficits, and aphasia can occur. Asymptomatic patients are rare. Seizures or epilepsy can occur at any stage of the disease (including as a presenting symptom) but are more often in the advanced stages [5].

GBMs can be either primary, mostly affecting older patients, or secondary, more common among young patients. Patients with secondary GBM have a better prognosis than those with primary GBM. DNA profiling revealed that these two subtypes are not histologically distinguishable [6]. Genetic alterations leading to primary GBMs include amplification and/or mutation of *epidermal growth factor receptor (EGFR*) (in chromosome 7q), homozygous deletion of the *cyclin-dependent kinase inhibitor 2A (CDKN2A-p16INK4a)* gene in chromosome 9p, amplification of the *mouse double minute 2 homolog (MDM2)* oncogene, mutations of the *neurofibromin 1 (NF1)* and *phosphatidylinositol 3-kinase regulatory subunit alpha (PIK3R1)* genes, and loss of heterozygosity of chromosome 10, including deletion of the *phosphatase and tensin homolog (PTEN)* gene. In contrast, the ones leading to secondary GBM are *tumor protein 53 (TP53)* (chromosome 17), *isocitrate dehydrogenase 1 (IDH1)*, and *isocitrate dehydrogenase 2 (IDH2)* mutations [7].

GBMs can also be divided into four subtypes based on transcriptional/RNA profiling, i.e., neural, proneural, mesenchymal, and classical. It is feasible to combine DNA alterations with subtypes defined by RNA expression. *IDH1*, *TP53*, the transcriptional regulator ATRX, *platelet-derived growth factor receptor alpha (PDGFRA), cyclin-dependent kinase 6 (CDK6), cyclin-dependent kinase 4 (CDK4), MET* mutations, the glioma CpG island methylator (G-CIMP) phenotype, and *NKX2-2*, *oligodendrocyte transcription factor (OLIG2)*, and *SRY (sex-determining region Y)-*Box 2 *(SOX2)* genes can be found in the proneural subtype. Mutation or loss of *NF1*, *CDKN2A*, and *TP53*, as well as the presence of molecular markers such as chitinase-3-like protein 1 (CHI3L1), MET, cluster of differentiation 44 (CD44), and proto-oncogene tyrosine-protein kinase MER (MERTK), can be found in the mesenchymal type. *EGFR* amplification, *CDKN2A-^p16INK4a^* deletion, and lack of *TP53* mutation can be detected in the classical type. Patients with the proneural subtype have a better survival rate despite little response to aggressive treatment, whereas patients with the classical or mesenchymal subtype benefit from aggressive treatment [8].

Apart from genetic alterations, epigenetic changes also play a crucial role in the pathogenesis of GBM. Epigenetic changes such as hypermethylation of the *O-6-methylguanine-DNA methyltransferase (MGMT)* promoter and other gene promoters (G-CIMP phenotype) are some of the earliest events in the evolution of tumors. In addition, *TP53* mutations occur early in low-grade gliomas and develop into secondary GBMs after *IDH* mutation (*IDH* mutation plays a prominent role in the G-CIMP phenotype development) [9,10]. In contrast, mutations and amplifications of receptor tyrosine kinase (RTK) genes (*EGFR*, *PDGFRa*, *KIT*, *MET*), *PTEN* loss, and *telomerase reverse transcriptase (TERT)* promoter mutations occur late in the development of GBM [11].

Overall, the most important alterations leading to GBM are *TP53*, *IDH*, *ATRX*, and *TERT* promoter mutations; *CDKN2A-^p16INK4a^* and *MGMT* promoter hypermethylation; PDGFR expression; and *EGFR* amplification and/or mutation [12].

### 2.2. Ependymoma

Ependymomas are rare neoplasms of the CNS that are derived from radial glial cells. They are more common in children than in adults. These tumors can be located in three different anatomical sites of the CNS. The first two sites, supratentorial (ST) and the posterior fossa brain (PF), mainly affect children. The spinal cord (SC) is the third most common organ affecting adults [13]. The symptoms may vary depending on the location of the tumor. In particular, the clinical course of ST and PF tumors may include headache, nausea, vomiting, vertigo, ataxia, gait disturbances, focal neurologic deficits, seizures, and hemiparesis [14]. In contrast, SC tumors present with sensory and motor deficits, lumbar and sacral pain, and bladder and intestinal dysfunction [15].

The WHO divides ependymomas into three grades according to histological criteria, i.e., grade 1 myxopapillary ependymoma and subependymoma (SE), grade 2 ependymoma, and grade 3 anaplastic ependymoma. However, these grades are insufficient to provide trustworthy information regarding the clinical course and disease outcome [16].

For this reason, studies have investigated the molecular classification of ependymomas, which can be a valuable prognostic tool. Based on whole-genome DNA profiling, ependymomas can be divided into nine subtypes, i.e., three subtypes for each of the three anatomical regions where ependymomas can be located. Three subtypes include the tumors previously histologically classified as SE, ST-EPN-SE, PF-EPN-SE, and SP-EPN-SE. The other two ST subtypes include ST-EPN-RELA, which contains the *C11orf95-RELA* fusion gene, and ST-EPN-yes-associated protein 1 (YAP1), which contains a high frequency of YAP1-mastermind-like domain containing 1 (MAMLD1) fusions. The two remaining PF subtypes are defined by methylation profiling because they lack recurrent mutations. They include PF-EPN-A and PF-EPN-B, which are distinguished by global levels of histone H3 K27 trimethylation, are high in PF-EPN-B, and are absent in PF-EPN-A. The latter occurs mainly in infants, whereas PF-EPN-B mostly affects older children and adults [17]. SC ependymomas are also epigenetically divided through methylation groups into myxopapillary ependymomas, classic ependymomas, and SEs. They are also characterized by aneuploidy or tetraploidy, *NF2* gene mutation, and loss of chromosome 6q. Furthermore, recent studies have identified a rare SC ependymoma type containing *MYCN* amplification with a poor outcome [18]. Overall, patients in the ST-EPN-RELA and PF-EPN-A subgroups had a worse prognosis than those in the other seven ependymoma subgroups [13].

### 2.3. Astrocytoma

Astrocytoma is the most common type of glioma. It arises from astrocytes and affects primarily the brain and sometimes the spinal cord [19]. They are either encapsulated or infiltrative, indicating low- or high-grade malignancies, respectively.

Astrocytomas are divided into multiple categories, the most important of which are diffuse or non-diffuse and adult or pediatric. Non-diffuse neoplasms are less frequent and more circumscribed and mostly manifest in children. Their main types are pilocytic astrocytoma (WHO grade 1), subependymal giant cell astrocytoma (WHO grade 1), and pleomorphic xanthoastrocytoma (WHO grade 2 or 3). There are also subtypes with greater malignant potential [20]. Diffuse astrocytomas are classified depending on their mutations and histological features, according to the 2021 WHO grading system as grade 2, 3, or 4. The diffuse astrocytomas are *isocitrate dehydrogenase (IDH)*-mutant and present no 1p/19q codeletion. Wild-type *cyclin-dependent kinase inhibitors 2A and 2B (CDKN2A/B)* are classified as either grade 2 or 3 (depending on their mitotic activity and anaplasia), but necrosis or microvascular proliferation can also be classified as grade 4. If a homozygous deletion of CDKN2A/B is also present, the gene copy number alteration is classified as grade 4 [21].

Furthermore, grade 4 astrocytomas also include *IDH-*mutant astrocytomas and *IDH*-wildtype GBMs. The latter are 95% of grade 4 astrocytomas, while *IDH*-mutant astrocytomas constitute 5% and are more frequent in patients <55 years old [22]. Patients with *IDH* mutations exhibit less aggressive progression than those with *IDH*-wild-type gliomas, and IDH mutations play a crucial role in the pathogenesis of gliomas. These mutations are also quite similar; therefore, they are the targets of multiple therapeutic approaches [23]. For homozygous *CDKN2A/B* deletion, the loss of *methylthioadenosine phosphorylase (MTAP)* expression is a qualified alternative biomarker [24]. Moreover, heparan sulfate-glucosamine 3-sulfotransferase 1 (HS3ST1) and calponin 3 (CNN3) are overexpressed in astrocytoma, and mutations in *phosphatidylinositol-4,5-bisphosphate 3-kinase catalytic subunit alpha (PIK3CA)*, *PDGFRA*, and *MYCN* are generally associated with poorer prognosis in gliomas [25]. Mutations in *PIK3R1* and retinoblastoma pathway genes, as well as 7p gain, 10q loss, and mutation in the *TERT* promoter, are unfavorable in low-grade gliomas [26].

### 2.4. Oligodendroglioma

Oligodendrogliomas are diffuse, rare subtypes of gliomas that constitute approximately 5% of these CNS tumors. They resemble oligodendrocytes and grow mainly on the frontal lobes of the brain [27]. The tumor cells carry an *IDH* mutation because of a 1p19q mutation. They are either grade 2 or 3, according to their characteristics. Grade 2 gliomas are considered low grade, while grade 3 gliomas, also known as anaplastic oligodendrogliomas, are malignant and have different proliferation rates [28].

Although oligodendrogliomas typically have the best prognosis among diffuse gliomas, a decrease in *protein tyrosine phosphatase receptor type D (PTPRD)* and *contactin-associated protein 2 (CNTNAP2)* gene expression can indicate more aggressive tumor development [29]. Another indication of an oligodendroglioma’s malignant mutation is the expression of insulin gene enhancer protein 2 (ISL2) and other genes related to angiogenesis, such as CX3C motif chemokine receptor 1 (CX3CR1) [30]. Additionally, somatostatin receptor type 2 (SSTRTA2) expression might indicate a more favorable outcome in patients with anaplastic oligodendroglioma and is emerging as both a biomarker and a treatment target [31]. *SLAIN1, ankyrin repeat and BTB domain containing 2 (ABTB2)*, *tripartite motif containing 67 (TRIM67),* and *developmentally regulated GTP-binding protein 2* (*DRG2)* are also overexpressed, and *neurogenic locus notch homolog protein 1* (*NOTCH1)* is associated with a poorer prognosis [25,26].

## 3. Liquid Biopsy

Undoubtedly, early diagnosis is pivotal for cancer patient survival. The widely employed method for cancer diagnosis, staging, and prognosis is tissue biopsy. However, the invasive nature of some biopsy methods, especially in CNS malignancies, can lead to significant morbidity and mortality, limiting their feasibility for repeated procedures. Moreover, the inability of traditional biopsy methods to monitor therapeutic response in CNS tumors adds to the diagnostic challenge. As imaging, histological, and biochemical approaches fail to reach the desired diagnostic accuracy, diagnosing CNS malignancies becomes a burden [32,33].

Recognizing these limitations, liquid biopsy has emerged as a promising alternative for cancer diagnosis. Its less invasive nature, coupled with its ability to capture biomarkers from diverse tumor sites, makes it a valuable diagnostic tool. Unlike traditional biopsy in which samples are limited, liquid biopsy identifies biomarkers from the entire tumor landscape. CSF is a better source of biomarkers for CNS malignancies than plasma. The blood–brain barrier hinders the release of biomarkers into the bloodstream, whereas CSF is in direct contact with the neoplastic tissues that produce and release those molecules. Furthermore, plasma-circulating biomarkers can originate from tumors anywhere in the human body, compared to CSF biomarkers derived solely from CNS tissues [34].

## 4. Biomarkers in CSF

### 4.1. Circulating Tumor DNA

Circulating DNAs (cDNAs) are single- or double-stranded DNA fragments with a size of 140–340 bp that freely roam in body fluids after being released by normal cells. They are thought to be a remnant of cells undergoing apoptosis or necrosis [35]. Considering that, it is normal that in situations with increased cell death, such as inflammation or neoplasms, the levels of cfDNA in body fluids will also increase. In particular, cfDNA from tumor cells is the circulating tumor DNA (ctDNA). CSF has been identified as the best source of ctDNA for treating brain tumors (Table 1) [36]. However, certain limitations exist. Tumors located close to the ventricular system or the subarachnoid space, meaning they are in direct contact with the CSF, have higher levels of CSF ctDNA in comparison to tumors located away from CSF, where even the inability to detect ctDNA is possible [37]. The more malignant the neoplasm is, the greater the detectable level of ctDNA in the CSF. Patients with lower-grade tumors have lower CSF ctDNA levels, whereas people with high ki67 levels have higher CSF ctDNA levels. CtDNA levels are highest in GBM [38].

The reason why ctDNAs are so important is that they can carry gene mutations and epigenetic alterations present in the primary tumor. Therefore, they can be useful tools for the early diagnosis and monitoring of therapeutic responses. The methods used to analyze ctDNA include droplet digital PCR (ddPCR), Sanger sequencing, and next-generation sequencing (NGS) [39].

Juratli et al. [40] used ddPCR to demonstrate that *TERT* mutations can be detected in the CSF of GBM patients with a specificity of 100% and a sensitivity of 92.1%, indicating its use as a diagnostic and prognostic biomarker, as well as a biomarker for therapeutic response. These findings are consistent with other studies showing the potential clinical value of *TERT* mutation as a ctDNA biomarker in CSF [41,42,43].

Several research groups have studied *IDH* mutations. Fujita et al. examined CSF samples from 48 glioma patients using ddPCR and found that *IDH* mutations can diagnose oligodendroglioma, astrocytoma, and GBM [44]. Wang et al. and Zhao et al., with a sample of 17 and 24 patients, respectively, also noted the diagnostic value of *IDH* mutations as a diagnostic liquid biopsy biomarker in astrocytoma patients, especially younger patients [38,45]. Martínez-Ricarte et al. revealed that the coexistence of simultaneous *IDH* and *TERT* mutations with the absence of *ATRX* and/or *TP53* mutations was present in patients suffering from oligodendroglioma. The 1p/19q codeletion characterizing this type of tumor was later confirmed by FISH [42].

A study by De Mattos-Arruda et al. reported that CSF was more sensitive than plasma ctDNA for identifying genetic alterations associated with CNS malignancies [46]. They sequenced the DNA of 23 patients with various brain tumors (primary or metastatic) and found that their ctDNA samples harbored mutations in the genes *TP53*, *IDH1*, *ANK2*, and *EGFR*, which can be used as diagnostic and treatment response biomarkers. *EGFR* mutations were identified as both diagnostic and therapeutic response biomarkers. In one patient in the present study, the frequency of the presurgical allele of *EGFR* mutation in CSF was 80% and was undetectable after surgery, indicating its great utility as a tool to observe the response to treatment [36].

Whole exome sequencing (WES) identified several gene mutations in the CSF of patients with ependymoma (*ANKS3*, *HIST1H3C*, *TTC16*, *MARS*, *CDH5*, and *COL6A1*), but the sample size was not large enough to reach statistical significance [36]. The other findings include *PIK3R1*, *CLCA3P*, *IDH,* and *PTEN*, *TERT*, and *TP53* as diagnostic biomarkers of astrocytoma and GBM, respectively. Using NGS, Cheng Lei et al. also identified several potential diagnostic and prognostic biomarkers for astrocytoma, namely, *H3F3A*, *TP53*, and *ATRX* [47].

**Table 1:** ctDNA biomarkers in the CSF for the identification of gliomas.

Biomarker (ctDNA)	Glioma Type	Methodology	Biomarker Value	Reference
**TERT**	Glioblastoma	ddPCR	Diagnostic, Prognostic, and Treatment response	[40]
**IDH1 mut, H3F3A mut, NTRK1, TP53, EGFR, ATRX, SMARCA4**	Glioblastoma	WES	Diagnostic	[41]
**TERT**	Glioblastoma	WES	Diagnostic and Prognostic	[41]
**PTEN, EGFR, CDKN2A loss**	Glioblastoma	NGS	Diagnostic	[38]
**TP53**	Glioblastoma Astrocytoma	NGS	Diagnostic	[38]
**IDH1**	Astrocytoma	NGS	Diagnostic	[38]
**FGFR1, APC, EGFR, RB1, SMAD4, ERBB2, KDR, IDH1, PTEN, TP53**	All Gliomas	NGS	Treatment response	[45]
**RB1, EGFR**	Glioblastoma	NGS	Diagnostic	[45]
**IDH1 mut**	Glioblastoma	NGS	Diagnostic	[45]
**EGFR**	Glioblastoma	NGS	Diagnostic, Prognostic, and Treatment response	[36]
**PIK3CA**	Glioblastoma	NGS	Diagnostic	[36]
**SMO**	Glioblastoma	NGS	Prognostic and Treatment response	[36]
**EGFR, TP53, IDH1, ANK2**	Glioblastoma	ddPCR	Diagnostic and Treatment response	[46]
**TERT**	Primary Glioblastoma	Sanger sequencing, ddPCR	Diagnostic	[42]
**IDH, TP53, ATRX**	Secondary Glioblastoma, Astrocytoma	Sanger sequencing, ddPCR	Diagnostic	[42]
**IDH, TERT + absence of ATRX and/or TP53**	Oligodendroglioma	Sanger sequencing, ddPCR	Diagnostic	[42]
**H3K27**	Midline Gliomas	Sanger sequencing, ddPCR	Diagnostic	[42]
**IDH**	Oligodendroglioma, Astrocytoma, and Glioblastoma	ddPCR	Diagnostic	[44]
**ANKS3, HIST1H3C, TTC16, MARS, CDH5, COL6A1**	Ependymoma	WES	Not a big enough sample	[43]
**PIK3R1, CLCA3P, IDH**	Astrocytoma	ddPCR	Diagnostic	[43]
**PTEN, TERT, TP53**	Glioblastoma	ddPCR	Diagnostic	[43]
**H3F3A, TP53, ATRX**	Astrocytoma	NGS	Diagnostic and Prognostic	[47]

### 4.2. MicroRNAs

Because of the great heterogeneity of gliomas, not all tumor cells exhibit identical DNA mutations. Consequently, analyzing RNA expression in biofluids might offer a more precise depiction of malignancy.

MicroRNAs (miRNAs) are small noncoding RNAs comprising 18 to 22 nucleotides. They are single-stranded and play important regulatory roles in the expression of most genes. This is accomplished by connecting to specific positions of the 3′-untranslated region of their mRNA targets, thus leading to mRNA degradation or reduced translation [48].

While typically intracellular, miRNAs can also be found extracellularly. There, they exist freely as circulating free miRNAs originating from apoptotic or necrotic cells or as part of a complex with the Ago2 protein. Furthermore, miRNAs can be secreted from cells inside exosomes [49].

It has been proven that miRNAs can act as tumor suppressors and promoters. In particular, activation of the NF-kappaB pathway and BCL2 protein, which occurs when certain miRNAs are absent, plays a major role in tumorigenesis [50]. Conversely, miRNA-10b and miRNA-21 are significantly elevated in gliomas, promoting oncogenesis. MiRNA-10b is the only miRNA completely undetectable in brain biopsies without any malignancy [51].

Studies have shown that miRNAs can be detected in all body fluids. However, in the case of brain tumors, CSF has proven to be the best reservoir of miRNAs. Plasma is also considered a good candidate because it allows easier and less invasive sampling than CSF, but it is not as reliable. The BBB and the high prevalence of miRNAs from other tissues outside the CNS may alter the plasma examination results, whereas CSF obtains miRNA molecules through direct contact with the tumor tissue [52]. Quantitative real-time PCR (qPCR) and NGS are employed to study the miRNA samples collected from CSF [53].

Therefore, miRNAs are considered potential biomarkers for diagnosis, prognosis, and therapeutic response monitoring and are the subject of research among various study groups (Table 2). Zeng et al. analyzed CSF samples from GBM patients who underwent treatment with temozolomide (TMZ) [54]. They found that patients with high levels of CSF miR-151a had a better prognosis after receiving TMZ therapy than those with miR-151a downregulation. During the CSF examination of 13 GBM patients and 13 nononcologic patients, the researchers distinguished the two patient categories by calculating the miR-21 levels in their CSF with a sensitivity of 87% and a specificity of 93% [55]. In CSF samples obtained from nononcologic patients, the miR-21 levels were 10 times lower than those in the GBM group [55]. These results are consistent with other studies that highlighted the value of miR-21, an antiapoptotic agent that is a biomarker for the diagnosis, prognosis, and monitoring of therapeutic responses [56,57,58].

An additional study recognizing high levels of miR-21 in GBM patients’ CSF revealed much higher levels of miR-21 in the CSF of patients suffering from various brain metastases, suggesting that miR-21 can also be used to help discriminate between primary and secondary brain tumors [59]. Furthermore, the combination of miR-10b and miR-196b was used as a prognostic tool; patients exhibiting elevated levels of this combination had a median survival period of nine months, whereas patients with low levels of the combination had a median survival period of 16.5 months. The different levels of let-7c, miR-140, miR-196a, miR-196b, and miR-10b in the CSF of patients can be useful in the differential diagnosis of GBM and other low-level gliomas (ependymoma, astrocytoma, oligodendroglioma).

MiR-30b-3p causes resistance to TMZ through complex mechanisms, suggesting its potential as a prognostic biomarker [60]. Geng et al. reported the diagnosis of GBM using miR-9 with an accuracy of 80%, and Qiu et al. showed that hypoxia-induced miR-1246 can help monitor tumor recurrence after chemotherapy because high levels of miR-1246 in the CSF can be detected in patients with tumor recurrence, contrary to pseudoprogression [61,62]. It has also been proposed that specific CSF miRNA signatures help differential diagnosis of CNS malignancies by evaluating the presence or absence of miRNAs such as miR-223, miR-711, miR-451, and miR-935 [63].

**Table 2:** miRNA biomarkers in the CSF for the identification of gliomas.

Biomarker (miRNA)	Glioma Type	Methodology	Biomarker Value	Reference
**miR-151a**	Glioblastoma	qPCR	Diagnostic and Prognostic	[54]
**miR-21**	Glioblastoma	qPCR	Diagnostic and Therapeutic response	[55]
**miR-10b,** **miR-21**	Glioblastoma	qPCR	Diagnostic, Prognostic, and Therapeutic response	[56]
**miR-200a, miR-200b, miR-200c, and miR-141**	Brain Metastasis	qPCR	Diagnostic, Prognostic, and Therapeutic response	[56]
**miR-30b-3p**	Glioblastoma	qPCR	Prognostic	[60]
**miR-21**	Glioblastoma	qPCR	Diagnostic, Prognostic, and Therapeutic response	[57]
**miR-451** **miR-223** **miR-711 and miR-451 + loss of miR-935**	CNS Tumors Glioblastoma	qPCR qPCR	Diagnostic Diagnostic	[63] [63]
**miR-10b + miR-196b** **let-7c, miR-140, miR-196a,** **miR-196b, and miR-10b** **miR-21**	Glioblastoma Glioblastoma, Astrocytoma, Ependymoma, and Oligodendroglioma Brain Metastasis	qPCR qPCR qPCR	Prognostic Diagnostic Diagnostic	[59] [59] [59]
**miR-9**	Glioblastoma	qPCR	Diagnostic	[61]
**miR-1246**	Glioblastoma	qPCR	Therapeutic response	[62]
**miR-15b and** **miR-21**	Glioblastoma	qPCR	Diagnostic	[58]

### 4.3. Circulating Tumor Cells

Circulating tumor cells (CTCs) are cells shed from solid tumors into various body fluids (blood, CSF, and urine). When these cells survive oxidative stress and attack the immune system, they drive tumor metastasis. However, their detection in biological fluids can provide useful information regarding their tumor of origin [64].

The isolation of CTCs is extremely difficult, especially given their rarity and heterogeneity. Most studies are limited to detecting CTCs in blood, while documentation regarding their presence in CSF is not comprehensive. To date, no CTCs have been isolated from the CSF of glioma patients. Nevertheless, CTCs have been successfully isolated from the CSF of patients with solid tumors metastasizing to the CNS. Researchers have achieved a diagnosis of leptomeningeal metastasis with sensitivities of 93% and 100% and specificities of 95% and 97.2%, respectively. Therefore, CTCs in the CSF are promising biomarkers for the diagnosis, prognosis, and monitoring of tumor progression in patients with brain metastases [65,66].

The method employed for identifying these cells is called CellSearch, which utilizes immunomagnetic enrichment to detect CTCs. It is based on targeting specific biomarkers on the cell surface. In particular, the membrane biomarker targeted is epithelial cell adhesion molecule (EpCAM) [67]. However, EpCAMs are expressed only in epithelial tumors such as breast, prostate, and colorectal tumors and not in gliomas or other brain tumors derived from neural tissue. Thus, CellSearch is unable to detect CTCs originating from CNS malignancies.

An alternative successful approach has also been described for isolating CTCs from GBMs. This method involves detecting the presence of glial fibrillary acidic protein (GFAP) on CTC surfaces [68]. CTCs have been detected in CSF and blood samples of 32 children suffering from various brain tumors using GFAP nanoparticles for magnetic separation [69]. Nonetheless, GFAP expression is not ubiquitous across all glioma cells and therefore is not an ideal biomarker for glioma diagnosis.

CTCs can be detected even in low-grade gliomas, such as ependymomas, oligodendrogliomas, and astrocytomas, except for GBM. Therefore, further investigations are imperative to explore novel techniques and potential biomarkers facilitating the isolation of CTCs from the CSF of glioma patients.

### 4.4. Proteins

The critical role of proteins in tumor survival and growth makes them important biomarkers with great potential as monitoring tools for gliomas (Table 3).

This could be true for tyrosine phosphatase receptor type Z1 (PTPRZ) protein and its soluble cleaved form (sPTPRZ). In a study on CSF samples from 86 patients with different glioma subtypes, schwannoma, multiple sclerosis (MS), or non-tumor disorders, elevated sPTPRZ and PTPRZ (and the corresponding mRNAs) were detected. The first was elevated in gliomas of all grades, and the second was diagnosed in glioma and schwannoma patients from the remaining brain [70]. Hori et al. demonstrated the potential use of IL-6 as a prognostic biomarker in GBM patients. Their study included 75 glioma samples, 54 of which were GBMs. They discovered a relationship between the expression of IL-6 and tumor infiltration from macrophages. This relation seems to be reflux since one induced another. Finally, high levels of IL-6 were correlated with a worse prognosis for GBM patients [71].

Two pairs of autoantibodies related to tumors that appear to distinguish GBMs from low-grade gliomas have been detected. These antibodies were against nucleolar protein 4 (NOL4) and Kalirin (KARLN) for GBMs and against UTP4 and coiled-coil domain-containing protein 28A (CCDC28A). However, the small sample size of 23 patients in this study prevents absolute certainty about the observations. Further research is needed to substantiate these findings [72].

Glial fibrillary acidic protein (GFAP), a structural astrocytic protein that differs according to the stage of the cell proliferation cycle, plays a notable role in multiple neurological disorders. Concerning gliomas, it is stated that the lower the levels of GFAP are, the higher the WHO grade of the gliomas. Furthermore, the serum levels of this protein could emerge as a potential biomarker [73]. It is suggested that the CSF levels of matrix metalloproteinase-9 could indicate a recurrence of a malignant glioma before any evidence is manifested in MRI [74]. A significant increase in apolipoprotein E (APOE) and apolipoprotein A1 (APOA1) protein levels in the CSF of GBM patients has been reported compared to those in the CSF of patients with low-grade gliomas [75]. Schmid et al. examined the role of chitinase-3 like-protein-1 (CHI3L1) and GFAP as potential CSF protein biomarkers in GBMs. They highlighted the diagnostic use of GFAP, since it was significantly lower than in controls, confirming the findings that have been previously mentioned. They also mentioned its use as a monitoring tool. CHIL3L1, which is associated with tumor aggressiveness and poor survival, is correlated with GBM volume, suggesting that it is both a prognostic biomarker and monitoring biomarker [76].

In an additional study, researchers measured the CSF and plasma MIC-1/growth/differentiation factor 15 (GDF15) levels of patients with intracranial tumors, most of whom were diagnosed with GBM, and compared them to healthy controls. They found that the CSF MIC-1/GDF15 concentration in patients with GBM was significantly greater than in controls and lower in those with newly diagnosed GBM than in those with recurrent GBM. Its elevated levels could be an aggravating prognostic factor [77]. The potential use of cyclophilin A (CypA) as a monitoring biomarker and possible therapeutic target in pediatric diffuse intrinsic pontine gliomas was supported by Saratsis et al. They also proposed using dimethylarginine dimethylaminohydrolase 1 (DDAH1) as a prognostic factor and indicator of glioma aggressiveness [78].

### 4.5. Metabolites

Glioma metabolites are another area of increasing interest concerning CSF biomarkers. Metabolites are a variety of molecules, intermediate or final products, of cell metabolism, many of which have been proposed as potential diagnostic or prognostic biomarkers of glioma growth. Many studies have proposed a plethora of diagnostic metabolites that can distinguish between malignant gliomas and healthy controls. The types of metabolites suggest certain metabolic routes that characterize malignant gliomas.

During the examination of the relationships between 124 metabolites and malignant gliomas, the CSF of 10 patients with malignant gliomas and 7 controls were analyzed. Among the 38 metabolites that were significantly more abundant in patients with malignancies was glycose-1-phosphate, glutamine, and 7-methylguanosine presented the strongest correlations [79]. In a study measuring 125 CSF metabolites, the significance of carnitine, 2-methylbutyrylcarnitine, and shikimate was underscored. The first two are associated with the lipid metabolism of GBM, while shikimate suggests a potential link between the tumor and the gut microbiome [80]. Interesting correlations were found between *IDH*-mutant and *IDH*-wildtype gliomas in a study concerning CSF CNS tumor biomarkers. Acetylcarnitine and shikimate levels were significantly greater in *IDH*-wildtype gliomas than in control CSF. Malic acid and succinate (involved in the tricarboxylic acid cycle) are also found in high concentrations in *IDH*-mutant gliomas and could serve as distinguishing factors from *IDH*-wildtype gliomas [81].

A novel study of CSF low-mass ions (LMIs) (10,408 candidates) from 32 primary brain tumor patients yielded interesting results. Its purpose was to distinguish between gliomas and nontumorous controls, gliomas of different grades, grade 4 GBMs, and medulloblastomas, as well as glioma patients with and without leptomeningeal metastasis. Grade 3 gliomas presented elevated sphingosine and ceramide levels, while in grade 4 gliomas, the highest LMI levels were those of glycol aldehyde, glyceric acid, and acetic acid. A total of 2674 LMIs could distinguish between gliomas and controls. Increases in the levels of carnitine and phosphate in GBM (among others) distinguish it from medulloblastoma, characterized by high concentrations of carboxylic acid and pteridine. Finally, according to the different techniques used, numerous LMIs could have a role in revealing the presence of leptomeningeal metastasis [82].

CSF metabolites were examined in 32 patients with glioma brain tumors, and the possible diagnostic and prognostic value of CSF metabolites in gliomas was investigated. Among the 61 metabolites identified, citric acid and isocitric acid concentrations were significantly greater in GBMs than in grade 1–3 gliomas. There were also more grade 1–3 *IDH*-mutant gliomas than *IDH*-wildtype gliomas. Furthermore, higher levels of lactic acid were related to a worse prognosis in patients with high-grade malignant gliomas. GBMs also present relatively higher levels of lactic acid and 2-aminopimelic acid than low-grade gliomas [83]. Zaeiner et al. studied the CSF penetration of regorafenib, a multikinase inhibitor, by its efficacy in patients with recurrent malignant gliomas. It reached low CSF levels and possibly produced certain growth patterns in the tumor, as indicated by the MRI, and the treatment response was poor in general. In conclusion, additional trials are needed to further investigate the prognostic relevance of regorafenib, and caution is advised in its use as a last-line treatment, as suggested by researchers [84].

### 4.6. Extracellular Vesicles

Extracellular vesicles (EVs) are cell-derived vesicles surrounded by cell membranes that carry various molecules to specific recipient cells. The three main EV types are microvesicles, exosomes, and apoptotic bodies. These differences arise from the different mechanisms of biogenesis, release, size, content, and their respective functions [85,86]. The EV content reflects the status of the original cell and contains molecular effectors that regulate and enrich the recipient cell, providing it with new growth “signals”. This is an important way for tumors to remodel their tumor microenvironment [87].

EVs have been widely examined as diagnostic and monitoring methods for gliomas (Table 4). The presence of the *EGFRvIII* mutation in glioma EVs was studied. This mutation constitutes a promising diagnostic tool. They found that, except for the abundance of wtEGFR RNA expression in the correlate vesicles, the *EGFRvIII* mutation proved to have 98% specificity as a biomarker for patients with GBMs [88]. Another promising EV biomarker for the diagnosis of GBM is miR-21. MiR-21 is highly expressed in GBM-derived EVs [89]. Moreover, significantly greater levels of *IDH1*-mutant mRNAs were detected in the EVs of GBM patients than in those of control individuals. The authors suggest that these findings could become an important tool for verifying the presence and type of glioma and for determining the most suitable tumor treatment [90].

A novel EV biomarker with promising results for GBM diagnosis and treatment was also studied. They detected significantly higher levels of miR-9 in EVs from GBM cells than in controls (as well as in the corresponding tissues). Interestingly, this biomarker was also more highly expressed in glioma stem cells and their EVs than in GBM cells, suggesting that EVs transfer miR-9 from the stem to GBM tumor cells. Additionally, the inhibition of miR-9 suppressed GBM malignant characteristics. Therefore, a new diagnostic and therapeutic method for GBM could be established [61].

Interesting findings revealed the ability of glioma cells to avoid the effects of therapeutic approaches. They showed that miR-1298-5p, a tumor progression suppressor, was excreted in large amounts per EV by glioma cells to myeloid-derived suppressor cells. In this way, not only was tumor growth not hindered but also suppressor cells were induced to resist anticancer immune activity. The prevention of the entry of this mRNA into exosomes could serve as a therapeutic target [91].

Subsequent studies have highlighted the complementary role of EVs in identifying biomarkers. These biomarkers, although not originating in CSF, can be easily extracted from the CSF of glioma patients. The overexpression of MYO1C in glioma EVs induced glioma cell migration, and its knockdown inhibited this effect. They also highlighted the greater suitability of CSF biopsy for this biomarker, which could provide better diagnostic and monitoring results. They finally proposed that, due to the high and problematic vascularization of GBM, targeting MYO1C could emerge as a therapeutic intervention that affects GBM blood vessels [92]. Regarding GBM therapy, promising experimental results on using the miR-30b-3p biomarker in hypoxic EVs have been published. Through these EVs, hypoxic glioma stem cells regulate the ability of GBM cells to develop resistance to TMZ. The two main findings of the research indicate that this biomarker could serve as a therapeutic target and that its concentration in CSF could determine whether GBM is TMZ-resistant [57].

Metabolic aspects of cancer progression have also been highlighted as a part of EVs implication in oncogenic mechanisms. Mechanisms that correlate obesity with cancer have been described, and recent studies reveal alterations in the nicotinamide adenine dinucleotide (NAD) metabolism in glioblastoma. In this context, visfatin, or else termed nicotinamide phosphoribosyltransferase (NAMPT), is a NAD-related metabolic enzyme and represents an abundant adipokine. NAMPT demonstrates increased blood-circulating expression levels linked to poor prognosis in patients with hepatocellular carcinoma [93]. EVs secreted by glioma stem cells are rich in NAMPT, and this NAMPT-enriched cargo is necessary for glioma stem cells to mediate sustained proliferation to radiosensitive cells. This mechanism could offer the potential of a new biomarker for predicting response to radiotherapy, which could be identified in the CSF of glioma patients [94].

## 5. Potential of Clinical Applications and Implications of Artificial Intelligence (AI)

The potential clinical implications of biomarkers identified in the CSF of glioma patients are promising, particularly in the context of drug resistance and disease monitoring. The comprehensive utilization of these biomarkers is crucial in advancing a more personalized approach to malignancies, thereby enhancing the efficacy of therapeutic interventions while minimizing adverse effects on patients. A small fraction of the numerous potential applications is described here. A topic of intense scientific interest is the GBM’s resistance to TMZ. Zeng et al. found that patients with higher levels of miR-151a in the CSF are more benefited from TMZ therapy since miR-151 targets XRCC4, not allowing it to facilitate the repair and survival of GBM cells [54]. Yin et al. worked on another biomarker that travels through EVs and promotes resistance to TMZ. They discovered that miR-30b-3p targets RHOB, resulting in the GBM’s resistance to TMZ [60]. Stringer et al. provide interesting insights regarding the role of CSF’s contents in inducing the tumor’s growth. They described the role of NUPR1, a transcription factor, which hinders the effects of TMZ and irradiation. They additionally found that trifluoperazine, an antipsychotic, was able to inhibit the role of NUPR1, proposing its use as a possible enhancing factor against the disease [95]. Geng et al. contribute to the research for personalized GBM treatment with their findings regarding miR-9. They claim that its inhibition could serve as an GBM cell suppressor since they deregulate the function of DACT3, one of the proteins responsible for the malignancy. They also suggest miR-9’s role as a promising diagnostic tool [61]. Another possible target is presented by Tian et al., who published that the knockdown of MYOC1 greatly effects the function of GBM’s blood vessels [92]. This is only a small proportion of the concurrent findings regarding CSF biomarkers and their possible personalized clinical use against gliomas, aiming at precise patient care.

Nowadays, research in the field of AI is advancing rapidly, focusing on innovations such as machine learning and deep learning. These developments can be applied in GBM diagnosis and treatment by assessing the vast quantity and complexity of biomarkers. In that way, algorithms can be created to help recognize specific biomarker patterns in each patient, leading to different prognostic and therapeutic decisions. Niu et al. selected some key differentially expressed genes and used the complement Naive Bayes algorithm for grades 2–3 and the random forest algorithm for grades 1–2 to predict the glioma stage with accuracies of 72.8% and 97.1%, respectively [96]. Gong et al. used 70 differentially expressed genes to predict the prognosis of glioma patients. They used machine learning to sort the patients into low- and high-risk groups and proved that the latter had a much worse overall survival [97]. In a similar study, Chen et al. selected differentially expressed genes and employed a random forest algorithm to determine gene importance. Following this, an artificial neural network was constructed to discriminate between different PANoptosis gene clusters, resulting in a highly accurate predictive model. The researchers also suggested that PANoptosis could be a potential therapeutic target [98].

AI can also assist in the identification of therapeutic targets for each glioma subtype. McInerney et al. analyzed over 20,000 genes from *IDH*-wildtype gliomas using TGCA data and an AI-driven algorithm. They managed to identify biological pathways important for the pathogenesis of gliomas. Furthermore, they discovered various diagnostic and prognostic biomarkers such as TSPYL2, JAKMIP1, and TMTC1. The presence of these biomarkers can alter therapeutic decisions and even serve as a target for novel therapies [99]. Therefore, by leveraging the ever-growing field of AI, research on glioma biomarkers can develop rapidly, moving one step closer to precise medicine for glioma patients.

## 6. Conclusions and Future Perspectives

The topic of non-tissue biomarkers for diagnosis, prognosis, monitoring, and therapeutic reasons regarding gliomas is an important domain of medical research, as proven by the number of relevant studies conducted in the last five years. This scientific interest reflects the need for invasive, tissue-specific monitoring techniques to be applied only limitedly. Developing specific alternative methods may reduce medical complications and the difficulties of invasive techniques. CSF, as indicated by numerous studies, could be a main source of information on CNS glioma progression. GBM seems to be the primary target of this novel approach.

Additional review and research articles discuss the necessity of biomarker identification in the CSF. The authors conclude that biochemical molecules secreted and detected in the CSF feature high specificity and chemical stability. Furthermore, this method offers the advantage of relatively easy repetitive monitoring of molecular alterations as a response to treatment. However, their unknown origin and other factors may affect their specificity [100]. Clinical utility of brain cancer biomarkers detected in liquid biopsies is presently restricted to the detection of *GFAP*, *MGMT*, and *IDH1* alterations in CTCs and ctDNA [101]. CSF biomarkers for monitoring brain cancer are being evaluated in ongoing clinical trials. In a phase I study (NCT03190967), CSF was collected at trial entry and after the end of cycle 2 of treatment for cell-free DNA analysis of T-DM1 and metronomic temozolomide for secondary prevention of HER2-positive breast cancer brain metastases. Cf-DNA sequencing detected 77 mutations in CSF, and 11 of those were uniquely identified in the CSF, therefore suggesting the presence of different clones during the development of brain metastases [102]. At this point, the NCT01106794 trial recruits pediatric patients to prospectively collect specimens with diffuse intrinsic pontine glioma or brainstem glioma, either during therapy or at autopsy, in order to characterize the molecular abnormalities of this tumor and compare RNA expression in tumor samples, normal brainstem tissue, and CSF. Similarly, the BrainChild-03 (NCT04185038) trial recruits patients with diffuse intrinsic pontine glioma to assess the administration of locoregional B7-H3 CAR T cells to children with recurrent/refractory CNS tumors and DIPG [103,104]. The NCT04692324 trial entitled “Cerebrospinal Fluid Biomarkers for Brain Tumors” is now recruiting patients with brain tumors to collect CSF for biomarker discovery and to evaluate the feasibility of serial CSF sampling for the assessment of tumor biomarkers.

For this method to be applied, new, larger, and more systematic clinical studies should be conducted. Additionally, certain questions should be answered by further research, for example, to determine the extent to which blood contents affect CSF and how the blood–brain barrier is affected by glioma. Furthermore, new noninvasive methods should be tested for their generalizability, feasibility, and potential dangers. Overall, research toward this goal is still in the early stages, but the published outcomes are encouraging for establishing a novel method for monitoring gliomas via CSF biomarkers.

## Figures and Tables

**Table 3:** Protein biomarkers in the CSF for the identification of gliomas.

Biomarker (Protein)	Glioma Type	Methodology	Biomarker Value	Reference
**PTPRZ** **sPTPRZ**	Glioblastoma, Astrocytoma, and Oligodendroglioma (Schwannoma) Glioblastoma, Astrocytoma, and Oligodendroglioma	Western blotting	Diagnostic Diagnostic	[70] [70]
**IL-6**	Glioblastoma	ELISA	Prognostic	[71]
**Autoantibodies against NOL4, KALRN, UTP4, and CCDC28A**	Glioblastoma and Low-grade Gliomas	Microarray analysis and Autoantibody screening	Diagnostic	[72]
**GFAP**	Astrocytoma and Glioblastoma	ELISA	Prognostic and Monitoring	[73]
**MMP-9**	Recurrent Malignant Gliomas	SDS-PAGE	Monitoring	[74]
**APOE and APOA1**	Glioblastoma and Low-grade Gliomas	MRM	Diagnostic	[75]
**CHI3L1** **GFAP**	Glioblastoma Glioblastoma	NanoESI-LC-MS	Prognostic and Monitoring Diagnostic and Monitoring	[76] [76]
**MIC-1/GDF15**	Glioblastoma	ELISA	Diagnostic, Prognostic, and Monitoring	[77]
**CypA** **DDAH1**	Pediatric Diffuse Intrinsic Pontine Glioma	MS	Monitoring and Therapeutic Prognostic	[78]

**Table 4:** Extracellular vesicle biomarkers in the CSF for the identification of CNS malignancies.

Biomarker (in EVs)	Glioma Type	Methodology	Biomarker Value	Reference
**EGFRvIII**	Glioblastoma	qPCR	Diagnostic	[88]
**miR-21**	Glioblastoma	RT-PCR	Diagnostic and Monitoring	[89]
**IDH1-mutant mRNA**	Glioblastoma	RT-PCR and ddPCR	Diagnostic	[90]
**miR-9**	Glioblastoma	RT-qPCR	Diagnostic and Therapeutic	[61]
**miR-1298-5p**	Gliomas	Whole-transcriptome sequencing	Monitoring and Therapeutic	[91]
**MYO1C**	Glioblastoma	LC-MS/MS	Diagnostic, Monitoring, and Therapeutic	[92]
**miR-30b-3p**	Glioblastoma	RT-PCR	Monitoring and Therapeutic	[60]

## Abbreviations

ABTB2	ankyrin repeat and BTB domain containing 2
APOA1	apolipoprotein A1
APOE	apolipoprotein E
CCDC28A	coiled-coil domain-containing protein 28A
CD44	cluster of differentiation 44
CDK4	cyclin-dependent kinase 4
CDK6	cyclin-dependent kinase 6
CDKN2A/B	cyclin-dependent kinase inhibitors 2A and 2B
CDKN2A-p16INK4a	cyclin-dependent kinase inhibitor 2A
CDNAs	circulating DNAs
CHI3L1	chitinase-3 like protein-1
CNN3	calponin 3
CNS	central nervous system
CNTNAP2	contactin associated protein 2
CSF	cerebrospinal fluid
CTC	circulating tumor cells
ctDNA	circulating tumor DNA
CX3CR1	CX3C motif chemokine receptor 1
CypA	cyclophilin A
DACT3	disheveled binding antagonist of beta catenin 3
DDAH1	dimethylaminohydrolase
ddPCR	droplet digital PCR
DRG2	developmentally-regulated GTP-binding protein 2
EGFR	epidermal growth factor receptor
ELISA	enzyme-linked immunosorbent assay
EVs	extracellular vesicles
EpCAM	epithelial cell adhesion molecule
GBM	glioblastomas
G-CIMP	glioma CpG island methylator phenotype
GDF15	growth/differentiation factor 15
GFAP	glial fibrillary acidic protein
HS3ST1	heparan sulfate-glucosamine 3-sulfotransferase 1
IDH	isocitrate dehydrogenase
IDH1	isocitrate dehydrogenase 1
IDH2	isocitrate dehydrogenase 2
ISL2	insulin gene enhancer protein 2
JAKMIP1	Janus kinase and microtubule-interacting protein 1
KARLN	Kalirin
LMIs	low-mass ions
MAMLD1	mastermind-like domain containing 1
MDM2	mouse double minute 2 homolog
MERTK	proto-oncogene tyrosine-protein kinase MER
MGMT	O-6-methylguanine-DNA methyltransferase
miRNA	microRNA
MS	multiple sclerosis
MTAP	methylthioadenosine phosphorylase
MYOC1	myocilin 1
NF1	neurofibromin 1
NGS	next-generation sequencing
NOL4	nucleolar protein 4
NOTCH	neurogenic locus notch homolog protein 1
NSPC	neural stem and progenitor cells
NUPR1	nuclear protein 1
OLIG2	oligodendrocyte transcription factor
PCR	polymerase chain reaction
PDGFRA	platelet-derived growth factor receptor alpha
PF	posterior fossa
PIK3CA	phosphatidylinositol-4,5-bisphosphate 3-kinase catalytic subunit alpha
PIK3RI	phosphatidylinositol 3-kinase regulatory subunit alpha
PTEN	phosphatase and tensin homolog
PTPRD	protein tyrosine phosphatase receptor type D
PTPRZ	protein tyrosine phosphatase receptor type Z1
qPCR	quantitative PCR
RHOB	ras homolog family member B
RTK	receptor tyrosine kinase
SC	spinal cord
SE	subependymoma
SOX2	sex-determining region Y-box 2
sPTPRZ	soluble cleaved form of PTPRZ
SRY	sex-determining region Y
SSTRTA2	somatostatin receptor type 2
ST	supratentorial
TERT	telomerase reverse transcriptase
TMTC1	transmembrane O-mannosyltransferase targeting cadherins 1
TMZ	temozolomide
TP53	tumor protein 53
TRIM67	tripartite motif containing 67
TSPYL2	testis-specific Y-encoded-like protein 2
WES	whole exome sequencing
WHO	World Health Organization
XRCC4	X-ray repair cross-complementing 4
YAP1	yes-associated protein 1
